# Chemsex and its implications for human immunodeficiency virus risk, testing, and prevention among men who have sex with men in Hong Kong

**DOI:** 10.3389/adar.2026.16956

**Published:** 2026-10-22

**Authors:** Alex Siu Wing Chan

**Affiliations:** Department of Social Work, Hong Kong Baptist University, Kowloon Tong, Hong Kong SAR, China

**Keywords:** chemsex, dating applications, HIV self-testing, human immunodeficiency virus, men who have sex with men

## Introduction

Chemsex defined as the use of psychoactive substances before or during sexual intercourse, represents a significant public health challenge among men who have sex with men (MSM), particularly in Hong Kong [1]. The practice has emerged as a critical factor amplifying the worsening human immunodeficiency virus (HIV) epidemic in this key population. Recent research reveals that approximately 6.9%–14.1% of MSM in Hong Kong report chemsex-related behaviours in their recent sexual encounters, with chemsex involving specific drugs such as methamphetamine, gamma-hydroxybutyrate (GHB), mephedrone, and ketamine affecting smaller but notable proportions of the community [2]. Online-dating MSM in Shandong Province demonstrate concerning patterns, with 37.8% perceiving their HIV infection risk as low despite engaging in high-risk behaviours, while 10.8% underestimate the harms associated with chemsex-related substance use [3]. This perspective aims to examine how chemsex among MSM in Hong Kong contributes to heightened HIV risk through its links with digital platform-facilitated partner seeking and broader psychosocial vulnerabilities. It also aims to highlight the implications of these patterns for HIV testing and prevention, and to argue for integrated, culturally responsive strategies that combine PrEP, HIV self-testing, behavioural support, and stigma-sensitive community interventions.

## Discussion

### Mechanisms linking sexualized drug use to HIV vulnerability

Chemsex increases human immunodeficiency virus vulnerability among men who have sex with men through interconnected behavioural, psychosocial, digital, and biological mechanisms. Psychoactive substances commonly used in chemsex, such as methamphetamine, poppers, ketamine, and other chemsex-associated substances, can reduce inhibition, impair judgement, and heighten sexual arousal, thereby increasing condomless anal intercourse, multiple and anonymous partnerships, and prolonged sexual encounters [4]. These risks are further intensified by geosocial networking applications and social media, which facilitate rapid partner-seeking, normalise chemsex, and expose users to pro-chemsex messaging.

At the psychosocial level, minority stress, stigma, concealment of sexual orientation, depression, and anxiety may increase susceptibility to chemsex while simultaneously weakening engagement in protective behaviours such as regular testing and consistent prevention practices [5]. Low perceived HIV risk and limited awareness of the harms associated with chemsex-related substance use may also reduce uptake of pre-exposure prophylaxis and timely HIV testing. In addition, these behavioural and psychosocial pathways may plausibly interact with biological processes, including mucosal injury, immune dysregulation, and inflammation, although direct evidence in Hong Kong MSM remains limited and further local research is required to confirm these mechanisms (Figure 1). These overlapping mechanisms position chemsex as a key driver of HIV vulnerability in this population.

**FIGURE 1:**
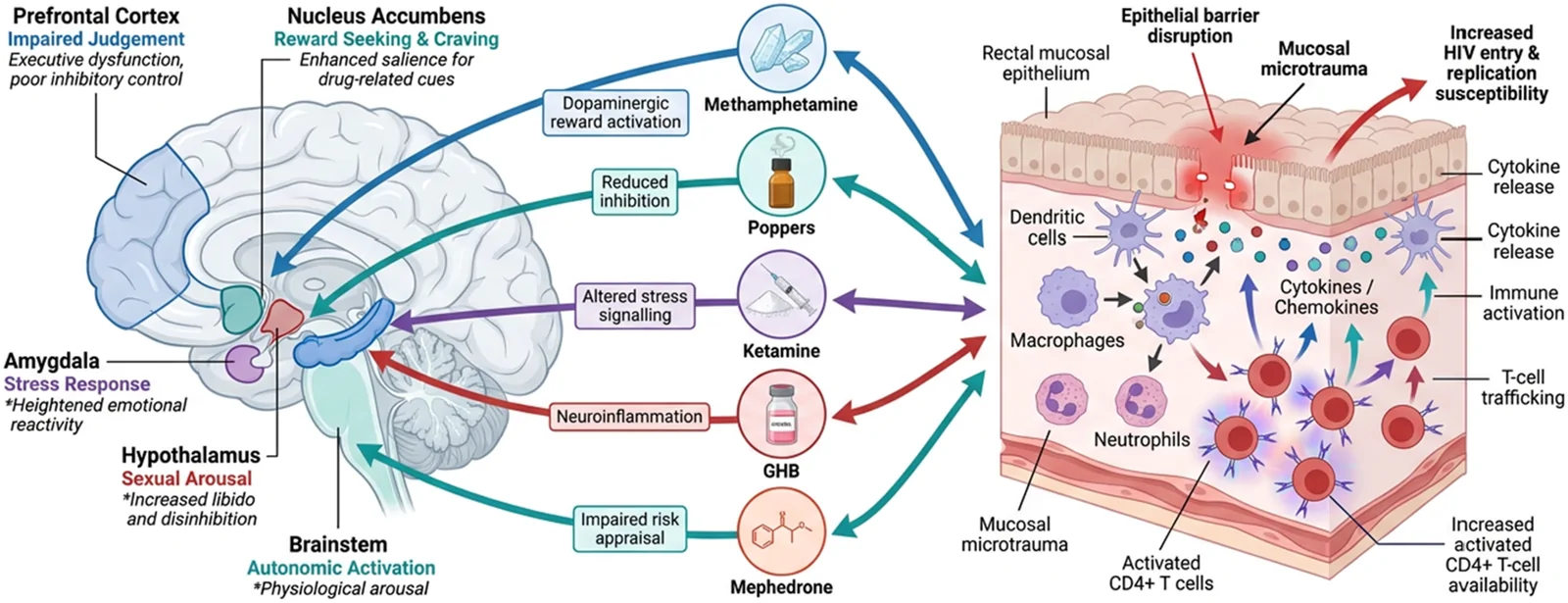
Neuroimmune and cellular mechanisms linking chemsex to HIV vulnerability.

### Specific substances and their health implications

The constellation of substances used in chemsex contexts includes primarily stimulants, disinhibitors, dissociatives, and anaesthetic-related substances [6]. For clarity, Hong Kong-specific evidence should be interpreted separately from evidence drawn from other jurisdictions. In Hong Kong, chemsex among MSM has most consistently been linked with methamphetamine, gamma-hydroxybutyrate (GHB), ketamine, and poppers, whereas studies from mainland China, Thailand, Japan, and the United Kingdom are used here as contextual evidence rather than direct local estimates. Among these substances, methamphetamine remains the most clinically important substance documented in Hong Kong, with associations with decreased condom use, multiple partnerships, and psychiatric comorbidity [5, 7]. In Hong Kong specifically, active psychotropic substance use among HIV-infected gay or bisexual men was associated with an approximately threefold increase in Axis I psychiatric disorders, with methamphetamine identified as the principal substance of concern [7]. Depressive disorders and psychotic disorders were also more common among those reporting active psychotropic substance use, particularly when methamphetamine use extended beyond chemsex contexts or became sustained over time [7].

In Hong Kong specifically, methamphetamine use was also linked to decreased condom use, with 30% of older MSM reporting recreational drug use [5]. Poppers, or alkyl nitrites, represent another prevalent substance associated with chemsex, with users showing higher likelihood of condomless anal intercourse and multiple sexual partners [4]. Although some evidence on poppers and other chemsex-associated substances comes from non-Hong Kong samples, these findings remain relevant because they describe behavioural mechanisms that are also observed in Hong Kong MSM, including disinhibition, condomless anal intercourse, and multiple partnerships. In addition, Hong Kong toxicology and emergency medicine evidence indicates that emerging ketamine analogues, particularly 2-phenyl-2-(ethylamino)-cyclohexanone, also known as 2-oxo-PCE, have appeared in local clinical and forensic cases, sometimes causing moderate to severe poisoning and requiring intensive medical care [8], the health implications of chemsex in Hong Kong should not be limited to HIV transmission alone, but should also include psychiatric morbidity, acute poisoning, and the need for coordinated HIV, mental health, emergency medicine, and substance use services.

### Social media and digital platforms as facilitators

Social media and gay social networking applications have emerged as primary vectors for promoting chemsex in Hong Kong’s MSM community. Hong Kong-specific prospective evidence shows that exposure to information supportive of chemsex on social media and gay social networking applications significantly increases subsequent engagement in chemsex among MSM [1]. The mediation pathway operates through Theory of Planned Behavior constructs, including positive attitudes toward chemsex, perceived support from significant others, and perceived behavioural control of refraining from chemsex [2]. This local evidence is particularly important because it demonstrates that digital platforms are not only neutral tools for partner seeking, but also social environments that shape perceived norms, acceptability, and self-efficacy regarding chemsex among MSM in Hong Kong.

Young MSM are particularly vulnerable, as platforms facilitate the normalisation of chemsex through influencer networks, peer-to-peer communication, coded language, and repeated exposure to substance-related sexual content [9]. However, evidence from the United Kingdom and other settings should be treated as explanatory rather than directly generalisable to Hong Kong, because digital cultures, patterns of disclosure, stigma, and service access differ across jurisdictions. In Hong Kong, where sexual minority stigma and limited disclosure of sexual orientation remain salient, dating applications may provide a relatively private pathway to sexual networking, but they may also intensify exposure to chemsex-normalising content and reduce opportunities for face-to-face risk counselling [1, 10].

### Sexual risk behavior and HIV transmission dynamics

Chemsex is closely linked to increased HIV transmission risk through multiple behavioural pathways. In Hong Kong, the central concern is not simply the presence of drug use, but the convergence of chemsex with condomless anal intercourse, non-regular or anonymous partnerships, app-mediated partner seeking, and inconsistent engagement with prevention services. MSM engaging in chemsex report substantially higher rates of condomless anal intercourse, particularly with non-regular and anonymous partners [11]. Although Cheung et al. [11] provides important evidence from Thailand and should not be presented as a Hong Kong prevalence estimate, it supports the broader biological and behavioural plausibility that chemsex can intensify HIV transmission risk through clustered sexual networks and repeated high-risk encounters. Similarly, evidence from London shows that MSM reporting chemsex had higher prevalence of condomless intercourse with serodifferent partners across HIV status categories [12].

These international findings are used here to contextualise the risk pathways observed in Hong Kong rather than to replace local evidence. Hong Kong’s compact urban environment, high mobile connectivity, and dense sexual networks may facilitate rapid partner seeking and repeated sexual contacts, while methamphetamine use in particular may enhance disinhibition, impulsivity, and prolonged sexual activity [5, 13]. Recent Hong Kong evidence also suggests a gap between HIV prevention awareness and actual protective practice, with PrEP use remaining uneven despite elevated risk among MSM involved in chemsex-related networks [14, 15]. The Hong Kong situation should be understood as a locally specific interaction between chemsex, app-mediated sexual networking, stigma, and inconsistent uptake of biomedical prevention.

### Complex psychosocial determinants

The emergence of chemsex among MSM reflects underlying psychosocial vulnerabilities rooted in minority stress, stigma, and unmet mental health needs [6]. For Hong Kong, these determinants require careful local interpretation because sexual identity concealment, family expectations, and social stigma may operate differently from Western settings where much chemsex literature has been generated.

Factors predisposing MSM to chemsex include non-acceptance of sexual orientation, guilt about sexuality, and depression scores indicating mental health distress [6]. In Hong Kong, social stigma, family pressure to conceal sexual orientation, and limited disclosure of sexual identity significantly shape substance use trajectories [10]. Enacted sexual minority stigma directly increases odds of transactional sex, injection drug use, and multiple partnerships [16]. Although Silvestri et al. [16] is not Hong Kong-specific, it supports the wider minority stress mechanism through which stigma can translate into sexual and substance-related risk.

Additionally, substance use patterns demonstrate strong associations with comorbid depression and anxiety, with 40% of older Hong Kong MSM reporting depression symptoms [5]. Hong Kong clinical evidence further shows that active psychotropic substance use among HIV-infected gay or bisexual men was associated with substantially increased psychiatric morbidity, particularly depressive and psychotic disorders, reinforcing the need to treat chemsex as both a sexual health issue and a mental health concern [7]. These findings indicate that interventions in Hong Kong should avoid framing chemsex only as individual risk-taking, because structural stigma, concealment, loneliness, psychological distress, and limited access to culturally safe services may all contribute to vulnerability.

### HIV testing and prevention service engagement

Despite elevated HIV risk, MSM with chemsex history often demonstrate paradoxical patterns of engagement with prevention services. Among MSM with recent chemsex in Hong Kong, pre-exposure prophylaxis (PrEP) uptake was 4.0% overall but 14.6% among those with chemsex experience, with 67.1% expressing willingness to use daily oral PrEP [2]. Perceptions related to PrEP, including positive attitudes, perceived support from significant others, and perceived behavioural control, significantly predicted willingness to use PrEP [1]. These Hong Kong findings suggest an important controversy for prevention policy: MSM involved in chemsex may have higher prevention motivation and greater contact with sexual health information, yet actual PrEP uptake remains constrained by cost, side-effect concerns, limited knowledge, stigma, and inconsistent service access. However, barriers persist. Perceived risk often remains low despite objective risk behaviours [17], and concerns about side effects, cost, and knowledge gaps limit PrEP uptake [18].

HIV self-testing offers a promising alternative to clinic-based testing, particularly when integrated with online real-time counselling support. In Hong Kong’s MSM community, 63.1% of experienced HIV self-testing users and 40.4% of new users completed HIV self-testing during implementation projects, demonstrating acceptability of home-based approaches [19]. For MSM who engage in chemsex, HIV self-testing may be particularly useful because it can reduce barriers related to stigma, confidentiality concerns, clinic attendance, and fear of judgement. However, testing frequency remains below national guidelines, with only 47.6% of HIV-negative MSM reporting more than one HIV test annually [12]. In Hong Kong, the implication is that HIV self-testing, PrEP counselling, mental health screening, and chemsex harm reduction should be delivered through integrated and low-threshold service models.

### Dating applications and behavioral risk amplification

Dating apps facilitate chemsex encounters by enabling easy partner discovery and coordination of high-risk sexual meetings [20]. A cross-sectional study found that among gay dating app users in Amsterdam, the proportion reporting chemsex engagement was significantly higher (29.3%) compared to MSM visiting STI clinics (17.6%). This suggests that geolocation-based dating apps directly connect users seeking chemsex partners [21]. Dating apps for MSM have facilitated unprotected sexual encounters and practices, with drug use during such encounters being widespread among MSM. The digital infrastructure of dating apps makes it easier to arrange chemsex sessions, sometimes involving specific substances like crystal methamphetamine, mephedrone, and GHB/GBL which users discuss openly on these platforms.

Geosocial networking applications constitute an important structural context influencing sexual health risks among MSM in Hong Kong and across Asia. Research indicates that app users are more likely than non-users to engage in condomless anal intercourse, recreational drug use before sex, and sex with multiple partners [3, 22–24]. In Hong Kong, dating applications have particular relevance because they can facilitate rapid partner seeking in a compact urban setting while also offering perceived privacy for MSM who may not openly disclose their sexual orientation. Moreover, the convergence of app use and chemsex may compound these risks, with app users demonstrating 1.01 to 1.22 times higher odds of associated sexual risk behaviours [25].

## Intervention and prevention strategies

Effective interventions require multilevel approaches addressing individual, interpersonal, digital, and structural factors. Theory of Planned Behavior-informed interventions targeting attitudes, social norms, and self-efficacy show promise in modifying chemsex behaviours [2]. Integration of behavioural health interventions into PrEP programmes significantly improves adherence and retention [26]. For MSM with chemsex history, brief motivational interviewing focusing on substance use reduction and sexual risk reduction demonstrates efficacy [27]. mHealth interventions, including apps providing education, medication reminders, and e-consultation, demonstrate high acceptability among high-risk MSM populations [28]. Nevertheless, international intervention evidence should be adapted cautiously for Hong Kong because service use is shaped by local factors, including stigma, confidentiality concerns, cost, family pressure, and the availability of community-based sexual health services.

In Hong Kong specifically, culturally tailored web-based interventions targeting MSM dating app users show potential in improving sexual health through promoting condom use, regular testing, and reducing chemsex [25]. Further Hong Kong intervention research has shown that internet-based approaches can reduce sexual harms associated with chemsex by improving self-efficacy and safer sexual practices among MSM [29, 30]. Community-based organisations must leverage social media platforms strategically to counter pro-chemsex messaging and promote combination prevention strategies. A clinically appropriate Hong Kong model should therefore combine PrEP, HIV self-testing, condom promotion, chemsex harm reduction, mental health screening, emergency referral pathways for acute intoxication, and stigma-sensitive counselling. This integrated model would respond to the reviewer’s concern by grounding recommendations in Hong Kong-specific evidence while using non-Hong Kong studies only to support broader mechanisms and intervention principles.

## Conclusion

This perspective identifies chemsex as an interconnected factor associated with HIV vulnerability among MSM in Hong Kong, linking digital platform-facilitated partner seeking, psychiatric morbidity, substance-related harms, and psychosocial vulnerability within one framework. Its key contribution is the localisation of chemsex-related risk to Hong Kong, where dense sexual networks, dating application use, stigma, limited sexual identity disclosure, and uneven engagement with PrEP and HIV self-testing may complicate prevention. Rather than viewing chemsex solely as a behavioural issue, this perspective frames it as a combined HIV prevention, mental health, toxicology, and digital health concern requiring coordinated, stigma-sensitive responses and further local research.
